# Universality and Cultural Diversity in Moral Reasoning and Judgment

**DOI:** 10.3389/fpsyg.2021.764360

**Published:** 2021-12-13

**Authors:** Lina Bentahila, Roger Fontaine, Valérie Pennequin

**Affiliations:** Laboratory PAVeA, EA 2114, Department of Psychology, University of Tours, Tours, France

**Keywords:** universal moral, moral judgment, moral reasoning, cross-cultural research, WEIRD and non-WEIRD societies

## Abstract

Many theories have shaped the concept of morality and its development by anchoring it in the realm of the social systems and values of each culture. This review discusses the current formulation of moral theories that attempt to explain cultural factors affecting moral judgment and reasoning. It aims to survey key criticisms that emerged in the past decades. In both cases, we highlight examples of cultural differences in morality, to show that there are cultural patterns of moral cognition in Westerners’ individualistic culture and Easterners’ collectivist culture. It suggests a paradigmatic change in this field by proposing pluralist “moralities” thought to be universal and rooted in the human evolutionary past. Notwithstanding, cultures vary substantially in their promotion and transmission of a multitude of moral reasonings and judgments. Depending on history, religious beliefs, social ecology, and institutional regulations (e.g., kinship structure and economic markets), each society develops a moral system emphasizing several moral orientations. This variability raises questions for normative theories of morality from a cross-cultural perspective. Consequently, we shed light on future descriptive work on morality to identify the cultural characteristics likely to impact the expression or development of reasoning, justification, argumentation, and moral judgment in Westerners’ individualistic culture and Easterners’ collectivist culture.

## Introduction

Morality plays a fundamental role in the functioning of any human society by regulating social interactions and behaviors. The concept of morality denotes a set of values, implicit rules, principles, and long-standing, and shared cultural customs, drawn on the opposition between Good and Evil to guide social behavior (Haidt, 2007). Morality often means having to make the decision “What should I do?” by linking mental states (emotions, reasoning, and desire) and the subsequent action(s). Moral principles thus define the guidelines as to what an individual should do, or is allowed to do, both toward others and themselves, and this is in relation to socially constructed beliefs (Matsumoto and Juang, 2013). It is a common heritage that an individual acquires during their development, across different social environments. Consequently, morality is connected to culture. The notion of culture should be looked at according to the definition of Hong (2009), who describes it as “a network of understanding, made up of opinion-based routines, of feelings and interactions with other people, as well as a body of affirmations and essential ideas on aspects of life.” The individual’s environment establishes shared cultural knowledge, which brings about affective, cognitive, and behavioral consequences on morality. This link leads us to ask ourselves: to what extent does culture impact upon human morality? More specifically, are there cultural patterns of moral cognition in Westerners’ individualistic culture and Easterners’ collectivist culture?

In this review, we are going to discuss the current formulation of moral theories that attempt to explain cultural factors affecting moral judgment and reasoning. The distinction will be to contrast the cognitive-developmental and the social interactional approaches to the later spectrum of approaches that address intercultural variation in moral judgment. We will present in detail cross-cultural studies on moral judgment, which will allow us to better understand universal and societal aspects of morality. Finally, we will consider cultural models of moral cognition by identifying specific moral justification and argumentation in Westerners’ individualistic culture and Easterners’ collectivist culture.

## Going Beyond a Developmental Approach of Morality To Account For Intercultural Moral Variations

Numerous theories have shaped the concept of morality and its development by embedding it in the field of social systems and values of each culture.

The scientific psychological study of morality can primarily be traced to the influential moral constructivist theory of Piaget (Piaget and Gabain, 1965; Piaget, 1985) and theory on the development of moral reasoning of Kohlberg (1976). Those theories are shaped by a philosophic heritage, strengthened by a liberal and individualistic vision of morality, backed by the works of Kant (1765), Mill (1863/2002), and Rawls (1971). For these authors, morality is universal as it is based on rationality which, by definition, is shared by individuals everywhere. Both Piaget and Kohlberg have developed stage theories that show different reasoning about moral issues depending on the level of moral development. Kohlberg (1976) developed his stage theory of moral development based on work of Piaget (1932) and he conceptualized three levels of moral development, and each level contains two substages. First, the pre-conventional stage precedes understanding and acceptance of social conventions. It refers to heteronomous morality, whereby the individual obeys the rules for fear of being punished. Second, the conventional stage refers to autonomous morality and represents conformity to expectations and conventions of society and authority. Finally, comes the post-conventional stage, in which the individual formulates and accepts general moral principles, which are implicit to the rules, and whereby the individual independently faces social approval. The stages and substages of Kohlberg’s theory of moral development are shown in Table 1. The focal point of their research is to question whether the same stages of development can be found in all cultures (Piaget, 1977; Kohlberg, 1981). A meta-analysis of 45 intercultural studies across 27 countries (Snarey, 1985) examined the universal affirmation of Kohlberg’s theory. The hypothesis, according to which the development stages are invariable, was well supported when there was an accurate adaptation of the content and when the language of the interview matched that of the subject. The transversal and longitudinal results indicate the presence of the pre-conventional stage and the conventional stage in all of the studied cultures. Additionally, a more recent study (Gibbs et al., 2007) compared 75 studies across 23 countries and suggests that the first two stages of Kohlberg’s theory are universal.

**Table 1:** The stages and substages of Kohlberg’s theory of moral development.

Pre-conventional stage Individuals obey the rules for fear of being punished.	*Substage 1:* Obedience and punishment
	*Substage 2:* Individualism and exchange
Conventional stage Individuals conform to expectations and conventions of society and authority. They avoid blame and seek social approval.	*Substage 3:* Good interpersonal relationships
	*Substage 4:* Maintaining social order
Post-conventional stage Individuals formulate and accept general moral principles. Rights of others can override obedience to laws and rules.	*Substage 5:* Social contract and individual rights
	*Substage 6:* Universal principles

A significant criticism concerning this theory was put forward by Bukatko and Daehler (2003); it fails to address the measuring of moral values specific to the cultures. Kohlberg is neither interested in the content of morality, nor in the specific values which emerge from it. His concern is rather in the structure of moral development, by looking at how thoughts and reasonings are transformed throughout the different stages. Nevertheless, understanding the values of each culture is necessary to apprehend the development of moral reasoning. For instance, the beliefs of the Afar people in Ethiopia valorize polygamy with shared spouses, which play an essential role in their society, whereas this practice is considered as a moral transgression in Western countries. Further still, people from Asian cultures react differently to moral dilemmas compared to those who come from Western cultures. Indeed, Asian societies focus more on maintaining a harmonious social order (Dien Winfield, 1982). The development of moral reasoning theory does not account for these observations, while intercultural research shows that values and moral principles can influence moral structure.

Research has equally examined social interactional differences and similarities in terms of morality through the lens of the social domain theory (Turiel, 1983). This shows that morality is firmly rooted in the social systems and values of each culture. Turiel defines three domains; the moral domain, containing rules which protect and regulate the rights or the well-being of individuals. The conventional domain is linked to the understanding of social conventions and the rules which control social interactions. A third, the personal domain, determines personal decisions and choices (Bukatko and Daehler, 2003). Turiel’s work suggests that all individuals are able to get along, no matter their culture, even if they have a difference of opinion, for the reason that they can recognize the differences between the three domains and use the same distinctive criteria (Tostain, 1999). He, therefore, assumes that all individuals split morality into the same three domains. However, this is far from being always the case. Induced abortion is a striking example. Some believe that this is a personal choice, a private decision, whereas others, notably due to religious reasons (it is God who gives life, and it is He who taketh away), view it as a moral transgression. In this sense, the limits with Turiel’s model are that it leaves aside the issue of beliefs and ideas that supports how individuals assemble such content and pushes it into only one of the three domains. Other research reveals that the difference between morality and social conventions could not be as universal as the domain theory suggests. Study of Shweder et al. (1987) comparing Indian and American children, and study of Haidt et al. (1993) comparing individuals from Brazil and the United States, indicate that the moral domain is defined in a much broader sense in India and Brazil. Concerns regarding purity, spiritual degradation, and moral expectations of loyalty toward one’s social group, are the concerns that also arise within the moral domains. They observed that actions and rules considered as personal choices or social conventions by Western society are more “moralized” in India or Brazil. As such, Indians and Brazilians assimilate their conventions with a universal morality. The distinction put forward by Kohlberg and Turiel between morality and convention is therefore not applicable to these populations. Finally, Turiel and Kohlberg are doubly in agreement here. As Universalists, they believe that the framework of morality is the same in its outlines everywhere. As formalists, they are not sufficiently interested in the content of morality and believe that the subjects, whatever culture they may be, can agree insofar as they refer to the same moral reasoning (Tostain, 1999).

Kohlberg’s view predominated for the past several decades but recent theoretical developments in social and cultural psychology (Shweder et al., 1997; Haidt and Joseph, 2004; Curry, 2016) built on an evolutionary approach suggest that our behavior can be explained by internal psychological mechanisms. From that standpoint, the relevant internal mechanisms are adaptations and products of natural selection. Hence, rudimentary moral intuitions, such as harm aversion and reciprocity, go back to the very beginnings of human history. Moral evolved long before the emergence of our kind and serve the adaptive function of facilitating cooperation within groups and against enemies. Indeed, this evolutionary framework focuses on motivational orientations rooted in evolved unconscious emotional systems developed by experiences that predispose someone to react to an act or events in a particular way. It is suggested that evolutionary adaptations of species appear to regulate behaviors and promote individual welfare. As such, the moral principles of an individual are relative to the culture they belong to. According to cultures, the notions of Good and Evil are differently defined and lead to different values and principles. The same action could very well be considered as a serious moral transgression in some cultures and as a simple social misdemeanor in others (Shweder et al., 1987). From this perspective, morality is extended beyond intercultural differences. Each society has a moral system, dependent upon its beliefs, ideologies, and views of the world (Jensen, 2011). The history of societies has shown, for instance, that divorce, induced abortion, or more recently same-sex marriages, are perceived by some as a direct breach of morality, and thus must be avoided at all costs. Not everybody, therefore, has the same idea of the domains to which morality can be applied (Skitka et al., 2005). Haidt (2007) suggests the term “moral community” to characterize each group that shares the same values and moral norms; these groups also share the same ideas about how to enforce them.

Several models have been conceived to come up with a broader, and more structured, approach to morality. Shweder and Sullivan (1990) and Shweder et al. (1997), who carried out a series of ethnographic studies, mainly in the United States and India, noticed that moral judgments of Indians draw on social rules which are difficult to apply universally, and which are founded on social and religious rules. In the United States, on the other hand, moral judgments draw on more liberal social rules, founded upon individual rights, justice, and the principle of avoiding harm. From these results, Schweder talks about moral pluralism and develops a moral taxonomy which he considers to be universal with three main types of ethics “the big three” (three ethics approach). He defines them as three essential morals, each one conveying a particular vision of morality. Cultures differ from each other morally, depending upon the importance allocated to each one of the three ethics. The ethics of Autonomy aims to protect the individual, their liberty, their rights, to help them satisfy their needs, and to achieve their goals. The ethics of Community aims to protect the integrity of the group, its structure, its organization, its reputation, as well as the roles and the status of its members. The ethics of Divinity insists upon protecting the soul and the spirit, as well as all the spiritual aspects of the individual and their natural environment (Shweder et al., 1997). Several studies have looked at the use of the three ethics in India, Brazil, Japan, the Philippines, and the United States (Haidt et al., 1993; Rozin et al., 1999; Vasquez et al., 2001; Jensen, 2015). Generally, a pattern is noticed whereby Western countries more frequently use the ethics of Autonomy than the other two ethics, while Eastern countries present arguments based on Autonomy and Community. For Schweder, the Western hemisphere, taking into account its individualistic references, resonates with the ethics of Autonomy, while the Eastern societies, given the fact that they are based simultaneously on interdependence among individuals and beliefs of divine or natural law, mainly advocate the ethics of Community and ethics of Divinity. The limit of Kohlberg’s theory and Turiel’s differences between morality, convention, and the personal domain is that it restricts morality to the ethics of Autonomy by insisting upon justice and individual rights. This is limiting from an intercultural perspective, as they falsely reduce the moral field to values favored by Westerners. Thus, according to ideological contingencies, perhaps a historical interpretation would permit us to understand that each society only expresses a part of this universal morality. Depending upon their vision of the world, the individual could conform to either one, or all, of these three ethics, but to varying degrees.

Haidt and Joseph (2004) broadened the approach of the “big three” in terms of morality in their moral foundation theory. The motivation at the heart of their work is that most of the research in moral psychology concentrates on two aspects: Good/Evil or reciprocity/justice but does not take into account the cultural differences observed in the other moral domains. For example, among Westerners, emphasis is put upon compassion and fairness, whereas Easterners regard obeying authority, loyalty toward the group, and matters of purity as justifiable moral concerns (Graham et al., 2011). The moral domain was widened to include these concerns. Consequently, the common ground of all cultures is composed of five main moral principles, each one being characterized by an adaptive function having emerged over time (Graham et al., 2009; Haidt and Kesebir, 2010). These founding moral principles correspond to five psychological mechanisms underlying moral activity and behavior (Graham et al., 2009). The principle of not doing harm (Care/Harm) prohibits all forms of suffering caused voluntarily to others and underpins the values linked to protection of the people. The principle of equity (Fairness/Cheating) aims to regulate exchanges and relationships between individuals by means of the idea of reciprocity. The principle of loyalty (Loyalty/Betrayal) refers to maintaining cohesion at the heart of the group, by means of valuing the links which unite the individual to their group. The principle of authority (Authority/Subversion) is based on maintaining the hierarchical structure at the heart of the group *via* the respect of status, societal roles, and associated duties. The principle of purity (Sanctity/Degradation) aims to protect the individual from contamination of their bodies, but also their spirits, by means of valorizing self-control and spirituality. Those theorists recently tentatively added a sixth foundation, Liberty/Oppression, which refers to reactance and resentment toward those who limit one’s freedom (Graham et al., 2013). Because the majority of the research conducted to date has focused on the original five foundations, our discussion will focus on those. Characteristics of the five founding moral principles are shown in Table 2.

**Table 2:** The five founding moral principles and their characteristics.

Moral Foundations	Characteristics
*Non-harm*	Benevolence, kindness, sympathy, and compassion
*Equity*	Reciprocity, trust, and respect of individual’s rights
*Loyalty*	Commitment, loyalty, and patriotism
*Authority*	Obedience, discipline, and submission
*Purity*	Chastity, devotion, piety, and cleanliness

Graham et al. (2011) examined the intercultural differences of the moral foundations of participants coming from Eastern cultures of South Asia, East Asia, and South-East Asia, and of participants coming from the Western cultures of the United States, the United Kingdom, Canada, and Western Europe. Overall, participants from Eastern cultures obtained higher scores when considering principles of loyalty and purity compared to Western participants. According to the same authors, the differences center around loyalty and purity, which are justifiable when one considers the cultural differences in terms of collectivism (Triandis, 2001) and the link between purity and religious practice, in particular in South Asia (Shweder et al., 1997). Graham et al. (2011) suggest representing these five founding principles as five markers on a “moral equalizer scale,” with varying levels depending on the moral systems. It is, therefore, the prioritization of moral values stemming from these principles, which differentiates cultures and individuals. Throughout these analyzes, it is conceivable that a pluralist universalism has its place, meaning that we can simultaneously take account of universal moral concerns (such as the worry of personal integrity, dignity, right to life, and, more generally, human rights) and also of specific beliefs of each culture. These new guidelines show once again what Kohlberg neglected, namely the role that specific life experiences of individuals and cultural representations can play in the formation of morality.

Graham et al. (2009) then describe the principles of non-harm and equity as the individualizing foundation, because they are all linked to individual rights and that the individual is at the center of moral values. This foundation strengthens the groups and institutions by linking individuals to roles and duties to constrain their imperfect nature. It is vital as it permits some cultures to get rid of egoism by directly protecting the individual and by teaching to respect the rights of others. The moral foundation theory affirms that this foundation is particularly widespread in Western societies. They emphasize the importance of personal rights, justice, and caring about the well-being of individuals (Vauclair et al., 2014). Nowadays, we refer to them as being individualistic societies known as Western, Educated, Industrialized, Rich, and Democratic (WEIRD; Henrich et al., 2010; Atari et al., 2020). The traits of an individualistic culture are autonomy, personal success, and the pursuit of uniqueness.

Nevertheless, cultures do not limit their value to that of protecting the individual. For this reason, Graham et al. (2009) defined the binding foundation, corresponding to three other principles (authority, loyalty, and purity) that put people together as groups (Doğruyol et al., 2019). This foundation restricts the liberty of individuals in favor of promoting the interests of the group (Vauclair and Fischer, 2011; Vauclair et al., 2014). Above all, the function of morality is social; it contributes to the definition of a shared ideal to ensure a harmonious group life. Morality regulates individuals’ egoism by encouraging them to adopt behaviors that facilitate cooperation (Haidt and Kesebir, 2010). One mainly finds this foundation within Eastern societies, associating it to collectivist cultures, labeled non-WEIRD (known as oriental, less educated, less industrialized, quite poor, and non-democratic). Their modes of social organization are possibly close to those observed in the distant past in traditional societies. Collectivist cultures extol interdependence among individuals, conformity, and emphasize the needs of the group above the pursuit of individual goals.

Empirical results support the theory of division of the individualizing and binding foundations between individualistic and collectivist cultures (Graham et al., 2009, 2011). These foundations establish the moral system based on the idea that all intuitions and feelings induce judgments and moral arguments. Thereby, individuals, throughout their experiences and developments, rely more on one or another of these foundations and moral principles. Dependent on history, religious beliefs, social ecology, and institutional rules (like the structure of kinship or the economic markets), each society develops a moral system. This defines several moral guidelines among which one can find reciprocity of the group, protection, support given to others, and defense of the unity of the group but also self-preservation (Graham et al., 2016). Maxwell (2011) acknowledges the idea according to which cultural context can make one or several foundations salient, sometimes even antagonistic. It is therefore important to take into account these prototypical foundations when evaluating the moral identity of cultures. However, it should not be assumed that just because of their singular experiences, or their culture, individuals live in completely different moral universes. Morality does not get reduced to one cultural moral or social status; in effect, there are universal moral concerns. The inter-individual heterogeneity has to be considered as individuals do not passively comply with social constraints and dominant portrayals of their culture (Lloyd, 1992; Spiro, 1993). An individual cannot be in agreement with these traits. They can be confronted with multiple representations that span different cultures and against which they can choose to distance themselves.

There are more recent expansions of the evolutionary approach, such as the theory of Morality-as-Cooperation (Curry, 2016) that explains that morality is a collection of biological and cultural solutions to the problems of cooperation recurrent in human social life. This theory predicts seven well-established types of cooperation; helping family, helping group, exchange, resolving conflicts through hawkish and dovish displays, dividing disputed resources, and respecting prior possession. From this framework, seven types of morality were found; obligations to family, group loyalty, reciprocity, bravery, respect, fairness, and property rights. This theory provides more detailed coverage of the moral domain whereas moral foundation theory (Haidt and Joseph, 2004) proposes only five. Indeed, there is no foundation dedicated to kin or reciprocal altruism, or hawkish displays of dominance such as bravery or property rights. Nevertheless, the theory of Morality-as-Cooperation neglects the role of disgust in moral reactions, found in the principle of purity in the moral foundation theory. A recent study tested the Morality-as-Cooperation theory with the following hypothesizes: those cooperative behaviors are considered morally good whatever the culture they appear in, and these seven moral values are universal (Curry et al., 2019). To test this prediction, they investigated the moral valence of these seven cooperative behaviors in an ethnographic record of 60 societies. The ethnographic coverage was drawn from six regions of the globe (Sub-Saharan Africa, Circum-Mediterranean, East Eurasia, Insular Pacific, North America, and South America). The research found, first, that these behaviors were always considered morally good. Second, these morals were observed in the majority of the societies. There were no societies in which any of these behaviors were considered morally bad. And third, these morals were observed with equal frequency across all regions of the world; they were not the exclusive preserve of Western societies. They finally conclude that cooperation is always and everywhere considered moral with those seven cooperative behaviors which may be universal moral rules. This survey shows cross-cultural regularities with moral values that exhibit a multifactorial structure, varying on these seven dimensions.

As we have just seen, social and cultural structures (beliefs, symbols, and practices) shape morality in human societies. To this effect, the transgressive nature of an act strongly depends on an individual’s moral system and the moral principles that he valorizes the most. The moral norms (specifically culturally) of an individual are anticipated and expressed over the course of judgment and reasoning. It would be premature to assume that all psychological processes, even basic ones, are immune to experience and culture (Wang et al., 2016), even more so when one focuses on high-level processes such as moral reasoning and judgment. The cultural characteristics susceptible to having an impact upon the expression or the development of reasoning, argumentation, and moral judgment among WEIRD and non-WEIRD populations, should be defined. To do so, we will be looking at the cognitive processes which underpin morality in these societies.

## Cognitive Processes Which Underpin the Emergence of Moral Systems in Weird and Non-Weird Societies

The leading theories on moral judgment attempt to specify precisely the role that cognitive and emotional processes play in the elaboration of this type of judgment. Their focus is on knowing to what degree the moral or immoral nature established takes its origin from a logical and controlled reasoning, or an automatic and unconscious intuition.

Can reflecting on a moral question change one’s mind? Are societies amenable to moral reasoning? For decades, the field of moral psychology with Piaget’s and Kohlberg’s cognitive-developmental theories and Turiel’s social domain theory emphasized the role of reasoning in moral judgment. For them, the answer is “yes” because moral development is intricately connected to cognitive development, and subsequently, to the development of the capacities of reasoning. These models draw from a Kantian approach to morality. For Kant (1765), to know if an act is moral, one should question one’s reasons, and by reasoning, reach the conclusion that the action can be established as a universal law of nature. Generally speaking, moral reasoning can be considered as a systematic and reflected approach that allows individuals to make moral decisions. The process of moral reasoning consists of three steps: the first is the definition of the situation, the second is the analysis of the situation, and the third is the making of the decision (Lyons, 1983). Moral reasoning includes justifications made for and during a moral action (Royal and baker, 2005; Smetana, 2006). Individuals would thus be influenced by a controlled cognitive process, which is a conscious mental activity through which one evaluates a moral judgment. Moral reasoning is linked to the development of controlled processes, but they are compromised by cognitive biases, in particular, that of egocentricity (Fontaine and Pennequin, 2020). The moral level of an individual would depend upon their capacities of reasoning, and as such, non-WEIRD societies would have a lesser moral development compared to WEIRD societies (for a synthesis, see Snarey, 1985). This is because Western societies are founded upon a philosophical tradition that puts the focus on debating and reasoning. The latter plays a pivotal role in institutions, whether it will be concerning education, justice, or even politics (Lloyd, 1992; Peng and Nisbett, 1999). Institutionalization of education theoretically means that individuals within WEIRD societies are naturally programmed to reason in an abstract manner. Furthermore, middle- or higher-class parents in Western societies have numerous arguments with their children and wait for them to offer explanations (Mercier, 2016). All of these factors suggest that reasoning is a particularly valorized cognitive ability in Western societies and that it is conceivable that their moral level is therefore higher. Their particular cultural context seems to create conditions that favor the development of moral reasoning abilities and a motivation to enter into argumentative activities. Several empirical data do however throw doubt upon these theses.

First of all, Shweder et al. (1997) exhibit that conventional responses from individuals in non-WEIRD societies do not represent a simple upholding of their tradition (based on religious beliefs and original myths), but more likely conform to an alternative post-conventional abstract reasoning based on different premises. Their analysis, based on interviews, shows that Easterners’ collectivist culture does not refer to individual rights (based on the premise that the subject is autonomous and free) as do Westerners’ individualistic culture. To this effect, Easterners organize their lives around an idea instead: the expression of self, based on interdependence between individuals, and that the place and the obligations each one has in society grant access to a morally acceptable life. Their beliefs are based on the premise that certain traditions allow a superior moral order to be obtained. This moral order is, for instance, described within religious societies in holy texts and bestows an ultimate meaning to human life. If one refers to Schweder’s three ethics, regarding the ethics of Autonomy, the ideas relative to individual rights are comparatively less widespread in the morals of Eastern societies. Among the latter, moral discourses uphold the duties, not intending to protect individual’s rights, but by upholding the social order or for religious reasons (Hwang, 2015). For example, according to Islam, life on Earth is short and temporary, whereas life after death is eternal and perpetual. Those who dedicate themselves to charity will go to Heaven, whereas those who commit sins will go to Hell. Among Buddhists, one must adhere to five principles: do not kill, steal, lie, be lascivious, and do not eat meat. The violation of one of these principles can lead to automatic retribution from Karma in the next life. This type of moral discourse falls within the model of the Easterners’ moral systems, which implies the ethics of the Community or the ethics of Divinity, rather than the ethics of Autonomy.

Next, intercultural studies have observed that the difference concerning the moral level of the two kinds of societies is linked to the fact that individuals from collectivist cultures resort more often to conventional type arguments, whereas individuals from individualistic cultures rely more willingly on abstract principles (Tostain, 1999). Two interpretations will be highlighted to explain this difference. The first, equally shared by Kohlberg, is to say that individuals from Eastern societies have a lifestyle that impedes their moral development. For example, less education, rigid social structures, or even archaic beliefs which constrain an individual to access autonomy of reasoning, hinder one to develop a true morality of rights and principles. This explanation is refutable because as we have seen, moral judgments can be justified in different ways. WEIRD societies are more likely to call for abstract principles to justify a moral judgment (see Kohlberg’s post-conventional stage). The second explanation is to consider that there is an ethnocentric bias. The alleged universal morality of Kohlberg’s theory is typically a Western trait. They attempt to show that there are other morals, equally as sophisticated, but based on different principles, stemming from a different vision of relationships between the person and the society.

Empirical facts bring out the limits of the traditional rationalist theories, which give a predominant role to reasoning in moral judgment. More recent research critiques and emphasizes the strength and importance of emotionally based moral intuitions. With the framework of the social intuitionist model, Haidt (2001) proposes a set of casual “links” connecting three psychological processes: intuition, judgment, and reasoning. As a matter of fact, it operates a model in which judgment is not the product of conscious reasoning but a product of intuitionist cognitive processes that are automatic and unconscious. This approach builds upon the dual-process theory (Wason and Evans, 1974; Evans, 1989; Stanovich and West, 2008). It proposes that multiple independent but interacting processing systems underlie thought, judgment, and decision-making. Two types of different rationalities characterized by two systems exist. The System 1; namely the heuristic system, is influenced by the beliefs of the individual. It allows one to think, speak, reason, make a decision, and act adaptively to meet one’s objectives without looking for consistency. The System 2; namely the analytical system, allows one to think, speak, reason, and make a decision according to a hypothetic-deductive approach. In the social intuitionist model, moral judgment is therefore predominantly intuitive, firstly linked to the heuristic system. It leads the individual to evaluate if the action of a person is acceptable or not from a moral point of view. The “*post-hoc* reasoning” posits (contrary to traditional rationalist models) that one’s reasoning is driven primarily by one’s judgment, rather than the other way around. According to social intuitionist theorists, an individual knows immediately if his judgment unearths a morally acceptable or unacceptable act. An intuition that he calls “gut-feeling” is sensed quickly and is full of affect without the individual necessarily being aware of the reasons that have led him to such a judgment. This explains the reason why some people do not know how to spontaneously explain their judgments. Reasoning, a conscious, intentional, and controlled process through System 2, only happens “retrospectively,” once the person has to justify an action in a conscious manner (Hauser et al., 2007). For that to occur, he will then refer explicitly to moral intuitions which guided his judgment.

Haidt (2003) discusses innate and universal moral intuitions guiding moral judgments. He identifies five categories of intuitions (corresponding to the five moral principles) that individuals inherit, produced by means of natural selection at work throughout human evolution. These intuitions are developed according to an individual’s background and culture. This model includes processes of social influences linked to the formulation of moral reasoning or judgment. To this effect, throughout one’s development, an individual is influenced by several members of a group. By verbalizing them, these individuals can trigger the emergence of certain intuitions which are conducive to beliefs, values, and ideologies of the group. As such, they can influence the moral judgment of the person. For this reason, some intuitions are able to develop and expand, and others are inhibited. This explains why WEIRD and non-WEIRD societies do not judge and justify in the same way. Everybody has areas of moral concern developed by evolution, in which some intuitions are more predominant than others. Haidt even suggests that there is a critical period during childhood, beyond which the categories of non-developed intuitions are subsequently eternally forgotten.

An individual’s spirit has a morally diversified content that is specific to social experiences. Children actively form their moral understanding in a cultural context that uses stories to shape and guide the development of their particular moral principles (Haidt and Joseph, 2008). For instance, according to the culture, the definition of a human being varies. It depends upon these definitions whether one opts to grant, or not, rights to individuals throughout a moral judgment. If one poses the question: “is induced abortion morally acceptable?,” some societies do consider it to be acceptable as the fetus is not yet perceived as a complete human being. They justify their thinking on the basis that the fetus has not experienced social acknowledgment, a rite of passage, etc. Other societies which abide by respecting the individual, consider this act to be unacceptable and justify their thinking by the motive that the fetus is a human being who has the right to life, and thus see this act as murder. Moral judgment happens based on moral intuitions, linking up the perception of a model in the social world (often a value or a vice) to an appraisal. These are the elements of rapid mental structuring and are specific to a domain that strongly influences moral judgment (Haidt, 2001). To this effect, if the five intuitions are innate, individuals simply learn which event, in their culture, counts as an act of prejudice or injustice. For example, over recent years, Western societies (notably Americans) have become sensitive regarding the topic of sexual abuse of children, to such an extent that they are appalled by social behaviors which are completely normal in other parts of the world. These include the idea of making children sleep in the same bed as a parent of the opposite sex until halfway through their childhood (Shweder et al., 1995), or kissing genitalia of little boys as a sign of affection, as is done in some countries in the Middle-East. With regard to this moral concern, these Western societies then react quickly, automatically, and using emotions.

Each society, therefore, has immediate implicit reactions to stories of moral violations created by their beliefs, values, and social ideologies (Haidt, 2001). Emotional reactions, such as anger or guilt, can sway judgments and moral behaviors. Neurological and behavioral data back up the idea that emotions are essential for moral judgments. Huebner et al. (2009) suggested that moral judgment is moderated by a fast and unconscious process that acts upon causal-intentional representations. Individuals are sometimes able to know that an act is not correct, without having the capacity to explain why this is so (Haidt, 2001). We are therefore led to believe that moral reasoning is only one of the factors, certainly not the strongest, which influences judgments and moral behaviors. According to Matsumoto and Juang (2013), culture can affect emotions in many ways. Human beings are born with a range of basic emotions. These are universally expressed among all humans by facial expressions. However, culture creates rules, directives, and norms which regulate these emotions and influence the system of basic emotions to maintain social coordination. For example, individuals from collectivist cultures tend to include emotions in the evaluation of their social values, whereas individuals from individualistic cultures are likely to include emotions in their evaluation of the environment. Studies have revealed that moral violations are perceived as more or less severe depending upon the current emotional state of a person (Greene and Haidt, 2002; Greene et al., 2009; Horberg et al., 2011). Emotions amplify moral judgment, and each society expresses emotions differently depending on the moral concern in question. In the thesis of Alqahtani (2018) which compares a Saudi population representing a collectivist culture with a British population representing an individualistic culture, one can see that the two groups did not experience the same emotions during moral violations of the moral foundations (see theory of Haidt and Joseph, 2004). As a matter of fact, in the Saudi population, the non-harm and equity foundations triggered resentment. The loyalty foundation triggered sadness, resentment, and apathy. The authority foundation triggered resentment and apathy. Last, the purity foundation triggered disgust. Within the British sample, the non-harm foundation triggered anger. The equity foundation triggered anger and disgust. The loyalty foundation triggered sadness, anger, and apathy. The authority foundation triggered anger and apathy. Last, the purity foundation triggered disgust. Emotion has a link with environmental conditions; it can thus create a moral judgment as a result of a motivational process, such as values, beliefs, needs, and objectives (Blasi, 1999).

Haidt’s model is the first to have highlighted the place of intuitions and the role of these associated emotions in moral judgment. He is, nevertheless, the object of several critics. Approaching morality from the intuitionist perspective leads to consider conscious moral reasoning secondary to automatic and unconscious in moral judgment. It is also considered that the rational discourse of morality has no relevant impact on moral decision-making and solution-finding. In his reaction to Haidt’s emotional reductionism, Lind (2016) explains that moral judgment can be strengthened if moral reasoning is trained and re-trained repeatedly. Instincts, emotions, and intuitions may be an evolutionary legacy in the human mind, arising unconsciously. However, with experience, human beings learn and develop conscious tools to understand their natural impulses and navigate them (Nowak, 2016). Evolution has endowed humans with moral emotions (including empathy), but they need more advanced instruments to deal with the demanding social contexts in which decisions are required. Following intuitions and emotions is not enough; this is why moral reasoning matters, especially in social contexts. For Lind (2016), moral judgment competence is to be defined as “an ability to apply a certain moral orientation in a consistent (manner, as trained, developed, and trustworthy moral subjects) and differentiated manner in varying social situations.”

Haidt’s model does not explain the process at the origin of moral intuitions, by giving an extremely limited role to controlled processes (Mikhail, 2007; Waldmann et al., 2012). In fact, Haidt and Kesebir (2010) only touch upon the use of the heuristic system among implied cognitive processes during a moral judgment. They do not provide any further explanation detailing the cognitive processes underpinning moral intuitions. To plug this gap, Mikhail (2007) developed a model describing the different mental processes, which drive all moral intuitions. He thus describes three steps. The first process consists of developing a structured representation of the situation, integrating its timeline, its causal chain, the intentions of its contributors, its moral properties, and all of the implicit pertinent elements. The second process involves forming a structured description of the situation by linking all of the characteristics together to carefully separate the desired effects from any collateral effects. The third process consists of applying a certain tacit understanding, principles, and specific rules to the situation, to determine its moral acceptability. Mikhail, having been inspired by the works of Chomsky (1957) and Rawls (1971), suggests that this knowledge takes the shape of a universal moral grammar which gives the individual a form of stability and systematicity within their moral intuitions. He considers that morality has at its center a nucleus of rules or innate principles. This moral grammar allows the individual, over the course of his development, to integrate into the value system of his environment to internalize specific moral principles of his cultural universe (Mikhail, 2011). Societies have a unique and universal moral competence that emerges from underlying, subjacent, and unconscious cognitive processes.

To test the confirmation of the existence of a universal moral grammar, Hauser et al. (2007) bring experimental elements to the fore by evoking judgments and arguments of people confronted by the tram dilemma (Foot, 1967; Thomson, 1976). They posed a dilemma presented under the guise of two different versions to 5,000 subjects coming from 18 WEIRD and non-WEIRD countries, including young people and adults (13–70 years of age), men and women, religious people and atheists, as well as varying levels of education.

In the first version, the moral dilemma was presented as follows: *A tram is moving at a high speed on its track. Five workers are carrying out repairs on the track. On another track, onto which the tram could be redirected, a sole laborer is working. An employee from the tram company who is near the railway switch point and who understands the situation, could or could not, redirect the tram. If he does so, he would avoid the death of five workers and if he does not, he would avoid the death of one sole laborer. Does he have the moral right to redirect the tram onto the other track?*

In the second version, the dilemma is presented in the following manner: *A tram is moving at high speed on its track. Five workers are carrying out repairs on the track. John, who is passing on a bridge above the track, understands that he could stop the tram by throwing something big down onto the track. A pedestrian carrying a big bag is walking next to him. If he were to push him onto the track, he would instigate the stopping of the tram and would save the lives of the five workers. But consequently, the life of the pedestrian would be sacrificed. But if he does not do it, he would avoid the death of only one sole worker. Does he morally have the right to push the pedestrian off the bridge?*

Faced with the first version, 89% of the subjects judged the action of redirecting the tram to be morally acceptable. On the other hand, faced with the second version, 11% of the subjects judged the fact of pushing the pedestrian onto the track to be morally acceptable. In both cases, moral reasoning has a universal character because the results are independent of the level of study, religion, and culture. These results suggest the existence of a universal moral grammar (Hauser et al., 2007).

As we saw, the social intuitionist model offered by Haidt gives a limited place to controlled processes in moral reasoning (Paxton and Greene, 2010). Hence, these authors propose an alternative dual-process model according to which intuitions and reasoning are equal. Moral reasoning would occur more frequently. Its function is not only to justify moral judgment but also to counteract the primary intuition. To this end, several studies have shown that an individual engages within extensive moral reasoning when they become aware that their moral judgment could be deemed as being incorrect, and that they look to go beyond an implicit negative attitude (Paharia et al., 2009). The context (notably cultural) in which an individual finds themself can push them to be particularly rational or to re-evaluate their emotional reactions. This model shows that the individual can be sensitive to arguments presented to them, that they then integrate them into their reasoning, and following that, they will judge the moral acceptability of a situation differently. Moral judgment thus seems to be the product of both automated and controlled processes. It is the temporality of these processes that differs. Automated processes include processing emotional information and doing this quickly, whereas controlled processes include slower reasoning, giving the person time to have consciously obtained abstract information and evaluated it systematically. According to the argumentative theory (Paxton et al., 2012; Mercier, 2016), reasoning comes *ex post* to justify moral decisions which happen instinctively. These authors explain that the two situations of the dilemma are independently examined without seeking coherence. In this situation, the choice made by an individual is the one that is the easiest to justify in relation to mainly unconscious moral principles. During moral reasoning, each person is thus motivated by their moral system. Here, reasoning has an ecological function as individuals are led to defend an opinion that is influenced by society and conformity, acting as a means of persuasion.

## Social Justification and Argumentation in Weird and Non-Weird Societies

Justification processes are a uniquely human phenomenon. In almost every form of social exchange, humans constantly justify their behaviors to themselves and others. Moral choices can be justified in different ways. To explain a practice, one can invoke public opinion (“the majority of people find this practice good”), customs in the culture (“we have always done it this way”), an eminent authority (“our leader or God commands us to do this”), or principles resulting from personal reflection (“it is not good to make others suffer”; Tostain, 1999). Science, laws, moral dictates, religions, and philosophical beliefs can be seen as large-scale justification systems that provide individuals guidelines for socially acceptable or unacceptable behavior (Henriques, 2011). Henriques (2011) introduces the justification hypothesis to provide a framework for understanding human beliefs and values with a cultural level process. The justification hypothesis is part of a larger theoretical framework called the Tree of Knowledge System developed by Henrique. Justifications can be associated with what Dawkins (1989) calls a meme, which is a unit of cultural evolution. We can clearly envisage the evolution of such systems. There are three key elements of evolution: variation (different justifications are offered), selection (certain justifications are better at legitimizing action than others), and retention (selected justifications are stored and repeated; Schaffer et al., 2008). From those elements, new justifications systems emerge through the course of human evolution.

The justification hypothesis answers the question of why there is such a variety of types of justification systems. Henriques (2011) explains that WEIRD societies have distinct systems of justification. Religion is separated from the law, the government, Science, and all other cultural institutions. On the other hand, non-WEIRD societies do not necessarily distinguish religious worldviews, explanations of natural phenomena, and prescriptions for social conduct in their systems of justification. This can explain why moral justification, which involves a value, a principle, and a judgment, is not the same between WEIRD and non-WEIRD societies. Schaffer et al. (2008) argues forcefully for the utility of conceptualizing religion as a large-scale justification system. The individuals follow different fundamental purposes serving as differentiating forces in the justification systems. As such, the core of culture relies on the presence of large-scale justification systems to coordinate and justify human moral’s opinions and behaviors.

Arguments are the substantive reasons put forward to justify one’s moral choice or behavior. The power of argumentation during moral debates is not the same for all cultures. In fact, the nature or the types of arguments accepted or rejected varies depending on the social and cultural context (Mercier, 2016; Mercier et al., 2016). Members of WEIRD societies have attributed more value to argumentation in their institutions for a long time, whether it be regarding science, rights, or politics (Peng and Nisbett, 1999). They put relatively less emphasis on saving social harmony (Kim and Markus, 1999; Oetzel et al., 2001) than non-WEIRD cultures. This permits them consequently to have more confrontational and open debates (Mercier et al., 2015). Furthermore, in WEIRD societies, individuals are confronted by a variety of choices and views of the world. In such cultures, one can expect to defend one’s choices; since it is probable one will encounter people who make different choices (Schwartz, 2004).

As a rule, members of Eastern societies have much fewer choices: much fewer religions to choose from, much fewer products to buy, much fewer professions to choose from, much fewer people to visit, etc. (Levi-Strauss, 1966). This relative lack of choice results in a lighter burden of justification. Individuals from Easterners’ cultures, therefore, need to resort less to argumentation to justify their judgments or moral choices. They have less appreciation for argumentation and can be more reticent to engage themselves in moral debates on subjects, such as euthanasia, induced abortion, religion, divorce, education, etc. In fact, they adhere more strongly to their moral beliefs, and this often demonstrates the power of the institutions that they valorize and their impenetrability toward demonstrations and logical arguments. Furthermore, these societies attribute a symbolic value to their moral decisions, so that it is without a doubt more important to make a socially acceptable decision than an intrinsically correct decision. To this end, the links between the individuals are strong and as such, a person should priorities the interests of their group, in the opinions and beliefs they hold (Triandis, 2011). Norms, obligations, and duties linked to the objectives, the safety, and the harmony of the group guide the moral decisions of individuals.

However, we could envision a universality concerning the role of argumentation, no matter the culture. In this perspective, individuals would be confronted with the myside bias. The Argumentative theory describes it as a bias whereby individuals overwhelmingly produce arguments defending their preferred opinions (Mercier, 2010). Consequently, reasoning rarely allows individuals to rectify their erroneous intuitions. The myside bias can lead to over-confidence regarding moral choices (Koriat et al., 1980). This bias can be a cognitive response to a specific cultural environment in which argumentation is valorized and where it is particularly important to be capable of defending one’s point of view. As predicted, these characteristics are adaptative and frequently present among adults in WEIRD populations (Mercier and Sperber, 2017), but they are equally noticed in a culture that differs in many ways to WEIRD cultures, such as among the K’iche people in Guatemala (Castelain et al., 2015), and there is no solid proof of their absence in other cultures.

Argumentation has an adaptive function because it greatly facilitates communication. Thereby, a second characteristic of the myside bias can be highlighted: the capacity to reasonably evaluate other’s arguments, by rejecting the weak and accepting those which are sufficiently strong (Hahn and Oaksford, 2007). Sufficiently strong arguments can prevail and make an individual change their mind on an opinion. But during a moral judgment, the latter can be put at stake in the framework of a debate, as the (heuristic) intuition is too powerful and reasoning remains insensitive to all counter suggestions. It is like an impenetrable model (Fodor, 2008). Above all, reasoning remains motivated by the needs and the moral motivations of the individual.

## Conclusion

Morality is a necessary parameter in the functioning of all societies. It defines an ideal to strive for as well as limits one should not transgress. It guides the social behaviors of individuals and plays a part in maintaining cooperation and cohesion at the heart of societies. Recent socio-cognitive research brings to light an intuitive, universal, and emotional character of moral judgment. It also highlights the essential role of reasoning in enabling argumentation or inhibiting moral intuitions. Indeed, reasoning allows individuals to mobilize moral principles that may be used to override moral intuitions. The tendency to control one’s biased intuitions has become widespread due to social influences. Moral reasoning thus has a significant role in moral judgment, including the suspension of moral intuitions in the presence of justificatory reasons. This effect depends critically on the strength of the involved arguments, knowing that the types of arguments accepted or rejected vary according to the social and cultural context.

The moral system is organized around major principles. Depending on the culture to which one belongs those principles take on a different weighting. Heterogeneity accrued in societies implies the creation of a consequent number of groups that differ in their values and moral perspectives. This variability raises numerous concerns for moral science on the topic of norms, such as the objective criteria according to which one can assert that an action or a specific practice is moral or not. On a descriptive level, this variability offers numerous possibilities for moral psychology to identify the background, the sources, and the structures of moral lives of societies.

## Author Contributions

LB wrote the article and made a substantial contribution to the concept of the article. RF and VP revised the article critically for important intellectual content. All authors contributed to the article and approved the submitted version.

## Conflict of Interest

The authors declare that the research was conducted in the absence of any commercial or financial relationships that could be construed as a potential conflict of interest.

## Publisher’s Note

All claims expressed in this article are solely those of the authors and do not necessarily represent those of their affiliated organizations, or those of the publisher, the editors and the reviewers. Any product that may be evaluated in this article, or claim that may be made by its manufacturer, is not guaranteed or endorsed by the publisher.
